# Surgical configurations of the pectoralis major flap for reconstruction of sternoclavicular defects: a systematic review and new classification of described techniques

**DOI:** 10.1186/s12893-019-0604-7

**Published:** 2019-09-13

**Authors:** Jude Opoku-Agyeman, David Matera, Jamee Simone

**Affiliations:** 1 Department of Plastic Surgery, Philadelphia College of Osteopathic medicine, Philadelphia, PA USA; 2 School of Osteopathic medicine, Philadelphia college of Osteopathic medicine, Philadelphia, PA USA

**Keywords:** Sternoclavicular defect, Sternoclavicular joint, Pectoralis muscle flap

## Abstract

**Objectives:**

The pectoralis major flap has been considered the workhorse flap for chest and sternoclavicular defect reconstruction. There have been many configurations of the pectoralis major flap reported in the literature for use in reconstruction sternoclavicular defects either involving bone, soft tissue elements, or both. This study reviews the different configurations of the pectoralis major flap for sternoclavicular defect reconstruction and provides the first ever classification for these techniques. We also provide an algorithm for the selection of these flap variants for sternoclavicular defect reconstruction.

**Methods:**

EMBASE, Cochrane library, Ovid medicine and PubMed databases were searched from its inception to August of 2019. We included all studies describing surgical management of sternoclavicular defects. The studies were reviewed, and the different configurations of the pectoralis major flap used for sternoclavicular defect reconstruction were cataloged. We then proposed a new classification system for these procedures.

**Results:**

The study included 6 articles published in the English language that provided a descriptive procedure for the use of pectoralis major flap in the reconstruction of sternoclavicular defects. The procedures were classified into three broad categories. In Type 1, the whole pectoris muscle is used. In Type 2, the pectoralis muscle is split and either advanced medially (type 2a) or rotated (type 2b) to fill the defect. In type 3, the clavicular portion of the pectoralis is islandized on a pedicle, either the thoracoacromial artery (type 3a) or the deltoid branch of the thoracoacromial artery (type 3b).

**Conclusion:**

There are multiple configurations of the pectoralis flap reported in the English language literature for the reconstruction of sternoclavicular defects***.*** Our classification system, the Opoku Classification will help surgeons select the appropriate configuration of the pectoralis major flap for sternoclavicular joint defect reconstruction based on size of defect, the status of the vascular anatomy, and acceptability of upper extremity disability. It will also help facilitate communication when describing the different configurations of the pectoralis major flap for reconstruction of sternoclavicular joint defects.

## Introduction

The very reliable and versatile pedicled pectoralis major muscle (PM) flap is currently considered the work horse flap for soft tissue reconstruction of chest and sternoclavicular joint (SCJ) defects [1–3]. The flap’s blood supply is based on the thoracoacromial artery (TAA) and the sternal perforators from the internal mammary artery (IMA). The TAA has four described branches, the deltoid, pectoral, clavicular and acromial. Sternoclavicular defects can result from many etiologies including debridement after osteomyelitis and tumor resection [1–5]. The pectoralis major flap has been used to reconstruct these defects [2]. Resection of the manubrium and medial aspect of the clavicle results in substantial defects, as well as potentially exposed bone and/or blood vessels, making soft tissue coverage essential in wound healing [6–8].

Apart from the pectoralis flap, other flaps have been used for this purpose. The most common amongst these are the latissimus dorsi flap and the rectus abdominis flap. Free flap reconstruction has also been reported as part of the reconstructive ladder [9]. The pectoralis major flap is the first line flap due to its proximity to the defect and robust and predictable blood supply [10–12]. The latissimus dorsi flap is another option. It can be harvested as a muscle or musculocutaneous flap. The blood supply is away from the zone of injury and may not be injured during SCJ resection, However, compared to the pectoralis major flap, it is far from the sternoclavicular joint and its arc of rotation may limit it from reaching the defect [5]. The rectus abdominis flap is another flap that has been described in SCJ reconstruction. It is a robust flap with a lot of bulk it’s blood supply and the flap itself is away from the zone of injury (sternoclavicular joint). The main disadvantage of the rectus abdominis flap is related to its abdominal donor site morbidity including hernias and weakness [13, 14]. Free flaps can be used when no viable local or regional flaps are available [9]. However, the use of free flaps is associated with significant morbidity compared to PM flap including flap failure and the need for more intensive monitoring.

Over the years, there have been reports of different configurations of the pectoralis flap for sternoclavicular reconstruction. We reviewed the current literature to document the various configurations of the pectoralis major flap that have been described for sternoclavicular defect reconstruction. We propose a classification system for the flap configuration to facilitate better communication when describing these procedures and also provide a proposed algorithm for the selection of the appropriate pectoralis major flap configuration based on this classification.

## Methods

**Fig. 1:**
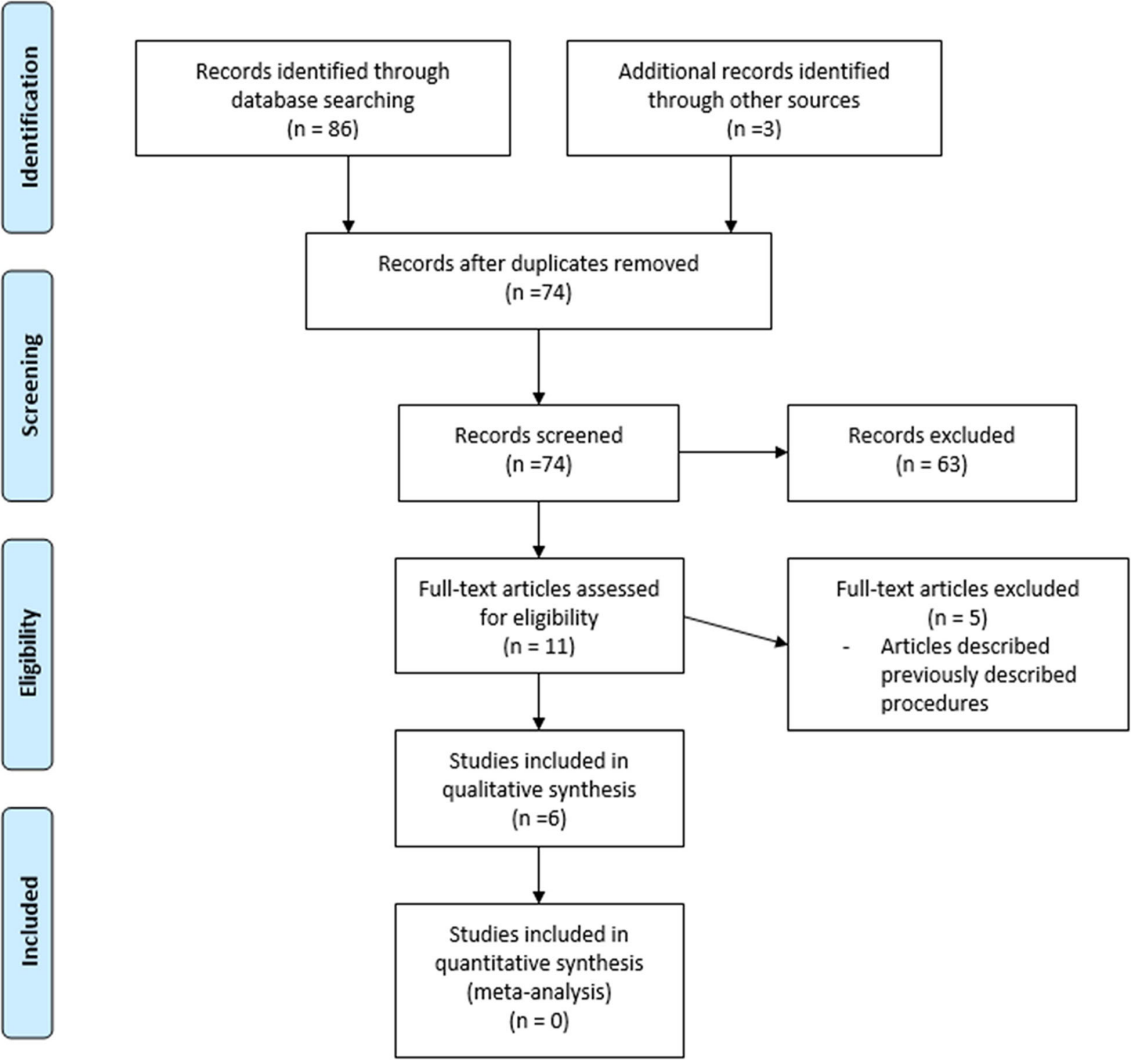
Flow chart of the literature search

We performed our systematic review in accordance to the guidelines set out in the Preferred Reporting Items for Systematic Reviews and Meta-analyses statement. We identified current published literature through a literature review. We did serial searches for articles published in English language. We searched Embase (up to 2019), PubMed (up to 2019), Cochrane library up to (2019) and Ovid medicine up to (2019). The search strategy included the following medical subject heading (MeSH terms): *Sternoclavicular defects; pectoralis flaps; sternoclavicular infections*; *sternoclavicular osteomyelitis; chest wound infection.* Related non-MeSH free-text search string was also included. Figure 1 illustrate our literature search strategy.

We included all full-text articles and abstracts with information on sternoclavicular defects, management of sternoclavicular joint defects and surgical management of sternoclavicular joint infection and tumors. All studies pertaining to the surgical management of sternoclavicular defects were included. The resulting articles were reviewed to select for papers that provide a description of the technique used for the reconstruction using the pectoralis major muscle flap. The first published paper describing the unique technique was included and duplicates excluded.

The articles were reviewed by and the techniques were catalogued. The images were reproduced by one of the authors. The techniques were then classified using our new classification system.

## Results

We identified 89 studies from our initial search. Only 11 of the articles provided a description of the technique involving the use of the pectoralis major muscle flap in the reconstruction of the sternoclavicular defects. Five (5) of the articles were excluded because they described the exact same procedures that has been previously described by a different author.

### Case 1

**Fig. 2:**
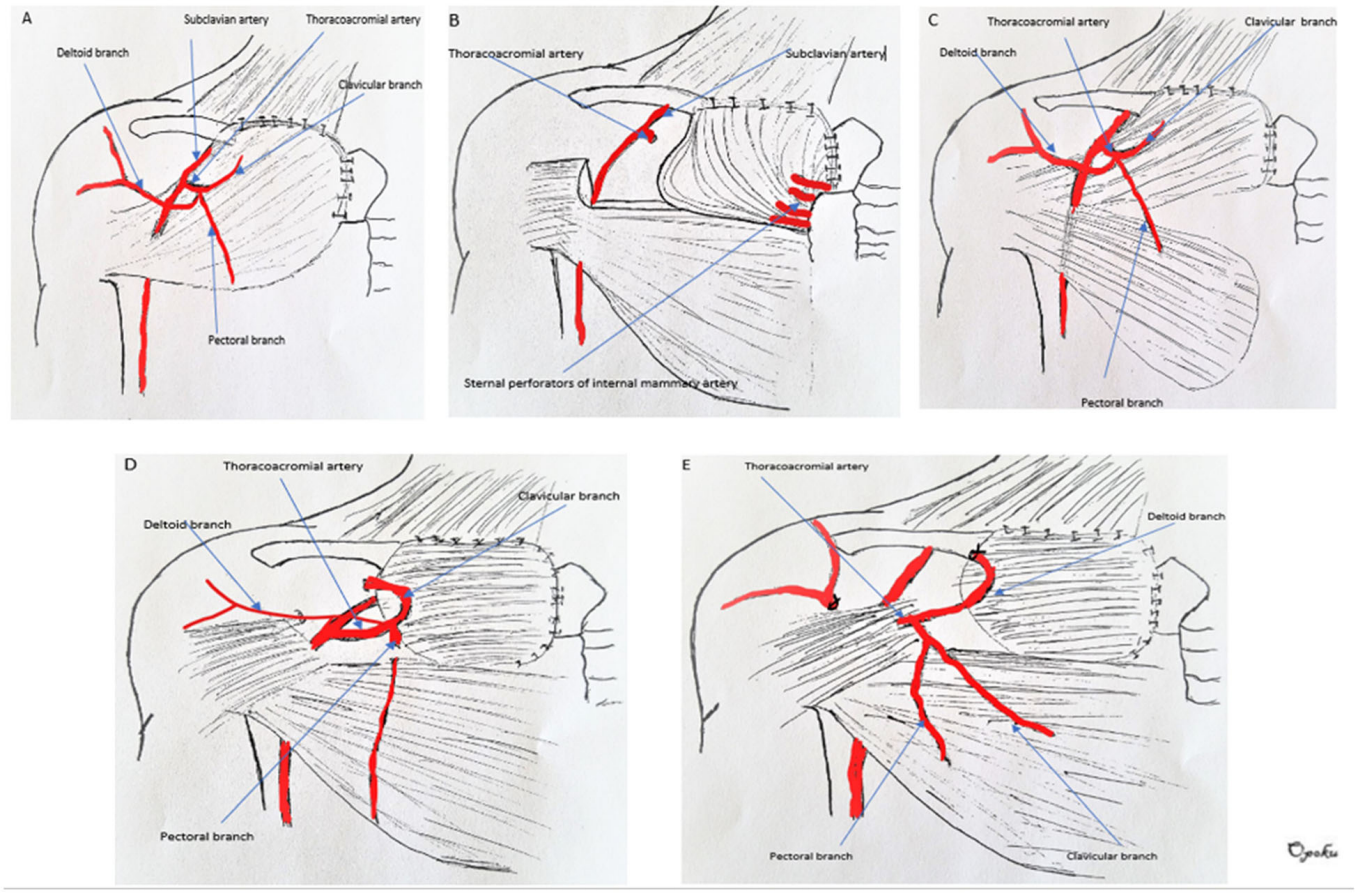
Different configurations of pectoralis major flap

The pectoralis major muscle advancement flap (Fig. 2a): The use of this flap for sternoclavicular defect reconstruction was first described by Munoz et al. in 1996 [15] and its modification, total release of humeral attachments by Opoku et al. in 2019 [16]. In this procedure, a flap consisting of skin and subcutaneous tissue is raised starting from an incision in the midline sternum. The extent of the flap is the deltopectoral groove. The pectoralis muscle flap is then raised from the chest wall to it attachment to the humerus making sure not to injure the TAA. This is done from medial to lateral chest wall. The muscle flap is then mobilized in a supero-medial vector to cover the sternoclavicular joint defect. If more length and muscle bulk is desired, the pectoralis muscle can be detached form it’s attachment to the humerus. In this configuration, the muscle is not split, none of the major branches of the Thoracoacromial artery is sacrificed, however, the pectoral perforator of the internal mammary are sacrificed.

### Case 2

Split pectoralis major muscle flap (Fig. 2b): First described by Zehr et al. in 1996 [1].

The SCJ defect is evaluated and the flap is planned. A flap consisting of skin and subcutaneous tissue is raised in a medial to lateral dissection. This dissection exposes the underlying pectoralis major muscle. An incision is made in the upper one-half of the pectoralis muscle at the lateral most aspect of the exposure. The fibers of the muscle are then divided in a longitudinal manner in the direction of the muscle’s origin on the sternum. The flap can then be rotated about 45 to 60 degrees to cover the SCJ defect. This configuration has ample muscle for soft tissue coverage. It is well vascularized from the intact sternal perforators of the IMA. The TAA is sacrificed.

### Case 3

Partial pectoralis major muscle advancement flap (Fig. 2c): First described by Song et al. in 2002 [17].

After SCJ resection, A flap consisting of skin and subcutaneous tissue is raised in the mid-sternum starting at the manubrium and carried caudally. The superior one third of the underlying pectoralis muscle is separated from the chest wall in a medial to lateral direction as far as the deltopectoral groove. The clavicular ant sternal attachments of the muscle is then released. The medial intercostal perforators are divided in the process. The muscle is then advanced medially to cover the SCJ defect. The resulting flap is a large flap with robust blood supply dependent on the TAA. The sternal perforators are sacrificed.

### Case 4

The islandized hemipectoralis major muscle flap (Fig. 2d): First described by Schulman et al. in 2007 [10]. After SCJ resection, a flap consisting of skin and subcutaneous tissue is raised exposing the pectoralis major muscle. The pectoralis is split at the demarcation between the clavicular and sternal portions. The muscle attachment to the clavicle and sternum are divided. The resulting clavicular portion of the PM muscle is reflected superiorly to expose the thoracoacromial artery. The muscle is then divided lateral to the TAA. This results in a clavicular portion of the PM that is completely islandized based on the TAA. The muscle is advanced supero-medially to fill the defect. This configuration has a small to moderate amount of muscle dependent on the TAA. It has a robust blood supply.

### Case 5

Deltoid branch-based clavicular head of pectoralis major muscle flap (Fig. 2e): First described by Al-Mufarrej et al. in 2013 [18]. It is basically a partial islandized pectoralis flap based on just the deltoid branch of the TAA. The branches of the TAA are not sacrificed.

After SCJ resection, the TAA is meticulously dissected out. The plane separating the clavicular and sternocostal portions of the PM is identified. The muscle is the split along this plane. The TAA pedicle and its branches are identified. The muscle fibers of the clavicular head of the PM are divided lateral to the pedicle. The artery is re-identified. The acromial branch of the deltoid artery can be divided to improve the muscle flap arc of rotation. Lateral to medial dissection in the subpectoral plane is performed as well as release of any sternal attachments. Once the muscle is islandized, the flap is used to cover the SCJ defect.

## Discussion

Sternoclavicular defects are rare in clinical practice. These defects are usually a result of surgical resection of the medial head of the clavicle and the manubrium for sternoclavicular joint infection or resection of tumors. These resulting defects are usually reconstructed with soft tissue. The pectoralis major muscle flap has been the workhorse flap for this type of reconstruction [10–12]. The first use of the pectoralis major muscle flap for reconstruction of chest defects was reported by Heuston in 1977 [19] and its first use in sternoclavicular defect reconstruction was described by Munoz [15]. Munoz essentially used the whole pectoralis major muscle as an advancement flap for the reconstruction of a sternoclavicular defect. The use of the whole muscle has been associated with loss of function of the pectoralis major muscle, aesthetic concerns related to the bulky appearance of reconstruction, and large access incisions. Since the use of the PM flap by Munoz in 1996, there have been multiple configurations of the PM flap to address these concerns. The various configurations have been termed differently in the reported literature for e.g., “compound pectoralis flap,” “split pectoralis flap,” “pectoralis advancement flap,” “islandized pectoralis flap,” etc. The names can be very confusing. For example, the islandized flap described by Schulmam and the deltoid branch flap described by Faisal et al. are both islandized flaps but differ based on the blood supply to the flap. There currently exists no classification system for the different configuration of the pectoralis flap for these reconstructions. We have classified the different configurations of the PM flap for sternoclavicular defects based on the reported cases in our literature review. Table 1 illustrates our classification system, the Opoku classification.

**Table 1:** Opoku Classification for pectoralis flap configuration for SCJ defect reconstruction

Classification	Description	Blood supply to flap	Example of flap
Type 1	Whole muscle advancement		
	With or without release of humeral attachment	TAA	Munoz et al. Opoku et al.
Type 2	Split muscle flap		
A	Advancement	TAA	Zehr et al.
B	rotated	Internal mammary perforators	Song et al.
Type 3	Islandized clavicular head flap		
A	Based on TAA	Whole TAA, distal TAA sacrificed	Schulman et al.
B	Based on deltoid branch of TAA	Deltoid branch of TAA	Mufarrej et al.

### Type 1: Whole muscle advancement

Type 1 configuration of the PM flap for sternoclavicular defect reconstruction includes procedures that use the whole pectoralis major muscle for reconstruction. It includes the pectoralis advancement flap in which the whole muscle is detached from its sternal clavicular attachments, mobilizing it laterally and advancing it medially to cover the defect [Fig. 2a]. This flap is based on the TAA. Included in this category is the flap when released from its humeral attachment to allow for more advancement.

### Type 2: Hemipectoralis muscle flap

Type 2 configuration includes splitting the pectoralis muscle and using the upper part of the muscle, usually the clavicular part for reconstruction. This configuration is subcategorized:

- Type 2A is a hemipectoralis rotated flap. In this configuration, the pectoralis muscle is split and the upper (sternoclavicular) portion is released from its insertion laterally. The flap is then rotated to fill the defect [Fig. 2b]. The flap is supplied by the internal mammary sternal perforators.
- Type 2B is a hemipectoralis advancement flap in which the upper part of the pectoralis major is split, and its sternoclavicular attachment is released. The muscle is then advanced to cover the defect. [Fig. 2c]. This flap is supplied by the TAA.

### Type 3: Islandized pectoralis flap

Type 3 configuration includes procedures in which a portion of the clavicular head of the pectoralis major muscle is split and then islandized by releasing all of its attachments.

- Type 3A is an islandized flap where the flap is supplied by the TAA. In this flap configuration, the distal part of the TAA is sacrificed [Fig. 2d].
- Type 3B is an islandized flap where the flap is supplied by the deltoid branch of the TAA. The TAA remains wholly intact without sacrificing distal blood flow [Fig. 2e].

**Fig. 3:**
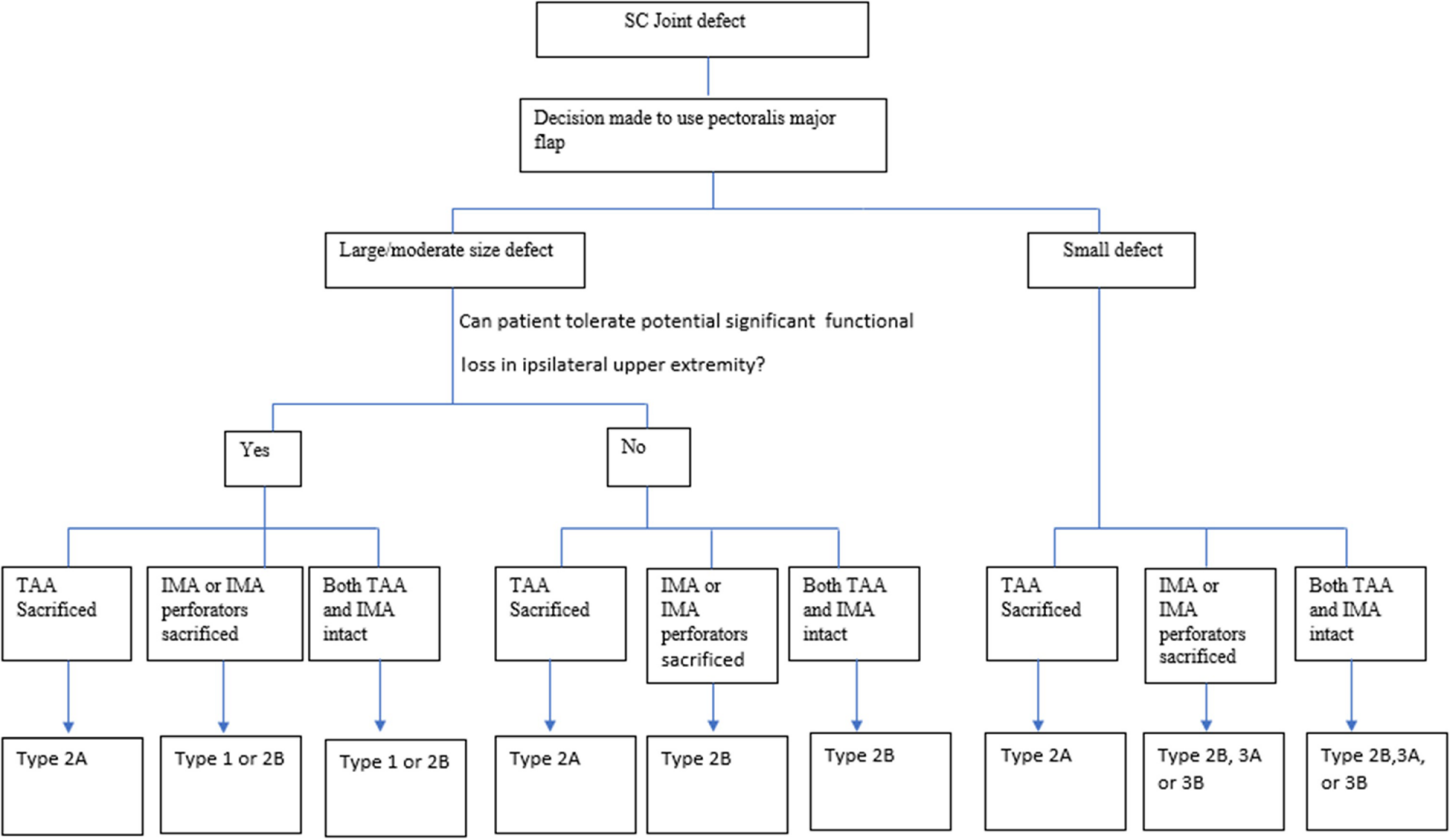
Reconstructive algorithm using pectoralis major flap for SCJ defect reconstruction

These different configurations have been described to address the different shortcomings of the other configurations. The general consideration of choosing the appropriate configuration once the decision has been made to use the pectoralis flap depends on the size of the defect, the status of the regional vascular anatomy and the functional consequences of the procedure on the ipsilateral upper extremity. For example, the internal mammary artery (IMA) may be sacrificed in tumor resection or may have been sacrificed in a previous procedure such as a coronary artery bypass graft. In this scenario, the flap variant that is dependent on the IMA perforators cannot be utilized. Some of the flap configuration have more bulk compared to the others any may be more suited for lager defects. The type 1 configuration and type 2 configurations uses advanced the whole muscle and about half of the pectoralis muscle respectively making them more suitable for large to moderate sized defects. On the other hand, the type 3 configurations us a portion of the clavicular portion of the pectoralis flap to provide coverage. This configuration has smaller bulk and may be more suitable for smaller defects. Some patients may be involved in activities or hobbies that require them to have intact upper extremity range of motion and full strength. Weakness in arm adduction associated with detaching the whole muscle origin or insertion may not be acceptable. This precludes the use of the type 1 configuration. A better choice will be another pectoralis flap variant where the pectoralis is left fully or partially attached to its insertion or origin. Based on these considerations we have proposed an algorithm for the use of the different pectoralis flap variant. Figure 3 illustrate our proposed algorithm. In our algorithm, when you have the choice of using a type 2 flap and both sternal perforators from the IMA and TAA are available, the Type 2B flap should be considered first since The TAA is a more reliable and robust blood supply.

## Conclusion

Sternoclavicular defects are rare in clinical practice. Different configurations of the pectoralis major flap have been described for this purpose mainly to circumvent the use of the entire muscle and limit the functional defects associated with the use the whole muscle. Our classification system, the Opoku Classification will help guide surgeons in the selection of the appropriate configuration of the pectoralis major flap for sternoclavicular joint defect reconstruction based on size of defect, the status of the vascular anatomy, and acceptability of expected upper extremity functional outcomes. It will also help facilitate communication when describing the different configurations of the pectoralis major flap for reconstruction of sternoclavicular joint defects.

## Data Availability

All data is contained within the published manuscript file.

## Abbreviations

IMA: Internal mammary artery
PM: Pectoralis major
SCJ: Sternoclavicular joint
TAA: Thoracoacromial artery

## References

[1] Zehr KJ, Heitmiller RF (1999). Split Pectoralis Major Muscle Flap Reconstruction After Clavicular-Manubrial Resection. Ann Thorac Surg.

[2] Joethy J, Lim CH, Koong HN (2012). Sternoclavicular Joint Infection: Classification of Resection Defects and Reconstructive Algorithm. Archives of plastic surgery.

[3] Shulman MR, Parsons BO, Lin H (2007). Islandized hemipectoralis muscle flap for sternoclavicular defect. J Elbow Shoulder Surg.

[4] Muñoz-Largacha JA, Slama J, Kalish J (2018). Approach to resection of sternoclavicular tumor abutting the common carotid artery in irradiated field. J Thorac Dis.

[5] Momeni A, Kovacj SJ (2016). Important consideration in chest wall reconstruction. J Surg Oncol.

[6] Allessi DM, Sercarz JA, Calcaterra TC (1988). Osteomyelitis of the clavicle. Arch Otolaryngol Head Neck Surg.

[7] Krespi YP, Monsell EM, Sisson GA (1983). Osteomyelitis of the clavicle. Ann Otol Rhinol Laryngol.

[8] Granick MS, Ramasastry SS, Goodman MA, Hardesty R (1989). Chronic osteomyelitis of the clavicle. Plast Reconstr Surg.

[9] Cordeiro PG, Santamaria E, Hidalgo D (2001). The role of microsurgery in reconstruction of oncologic chest wall defects. Plast Reconstr Surg.

[10] Li EN, Goldberg NH, Slezak S (2004). Split Pectoralis Major Flaps for Mediastinal Wound Coverage: A 12-Year Experience. Ann Plast Surg.

[11] Spiess Alexander M., Balakrishnan Chenicheri, Gursel Eti (2007). Fascial Release of the Pectoralis Major: A Technique Used in Pectoralis Major Muscle Closure of the Mediastinum in Cases of Mediastinitis. Plastic and Reconstructive Surgery.

[12] Ascherman JA, Patel SM, Malhotra SM (2004). Management of sternal wounds with bilateral pectoralis major myocutaneous advancement flaps in 114 consecutively treated patients: refinements in technique and outcomes analysis. Plast Recon Surg.

[13] Villa MT, Chang DW (2010). Muscle and omental flaps for chest wall reconstruction. Thorac Surg Clin.

[14] Skoracki RJ, Chang DW (2006). Reconstruction of the chest wall and thorax. J Surg Oncol.

[15] Gonzalez Munoz JI, Cordoba Pelaez M, Tebar Boti E, Tellez Cantero JC, Castedo Mejuto E, Varela de Ugarte A (1996). Surgical treatment of sternoclavicular osteomyelitis. Arch Brononeumol.

[16] Opoku-Agyeman J, Perez S, Behnam A (2019). Reconstruction of sternoclavicular defect with completely detached pectoralis major flap. Journal of Surgical Case Reports.

[17] Song HK, Guy TS, Kaiser LR (2002). Current presentation and optimal surgical management of sternoclavicular joint infections. Ann Thorac Surg.

[18] Al-Mufarrej F, Martinez-Jorge J, Carlsen BT (2013). Use of the deltoid branch-based clavicular head of pectoralis major muscle flap in isolated sternoclavicular infection. JPRAS.

[19] Hueston JT, McConchie IH (1968). A compound pectoral flap. Aust NZ J Surg.

